# Current state of evidence of cannabis utilization for treatment of autism spectrum disorders

**DOI:** 10.1186/s12888-019-2259-4

**Published:** 2019-10-29

**Authors:** Rumi Agarwal, Shanna L. Burke, Marlaina Maddux

**Affiliations:** 1 FIU Embrace Florida International University, 11200 SW 8th Street, Miami, FL 33199 USA; 2 BRAINN Lab, Florida International University School of Social Work, 11200 SW 8th Street, Miami, FL 33199 USA; 3 Florida International University School of Public Health Robert Stempel College of Public Health and Social Work Health Promotion and Disease Prevention, 11200 S.W. 8th Street, Miami, FL 33199 USA; 4 Florida International University School of Social Work Robert Stempel College of Public Health and Social Work, 11200 S.W. 8th Street, AHC5 585, Miami, FL 33199 USA; 5 Easterseals Blake Foundation, 750 E Broadway Blvd, Tucson, AZ 85710 USA

**Keywords:** Autism spectrum disorders, Autism, Cannabis, Cannabinoids

## Abstract

The core symptoms and co-morbidities associated with autism spectrum disorders (ASD) affect daily living and quality of life. Existing pharmacological interventions are only able to attenuate some related symptoms but are unable to address the underlying etiologies associated with ASD. Anecdotal evidence, which claims benefit from the use of cannabis to treat symptoms among this population, has been gaining popularity as families seek solutions.

This paper analyzed recent peer-reviewed literature to identify the current state of evidence regarding cannabis use for the ASD population. Systematic reviews, reports, and experimental studies were assessed to understand the current extent and nature of the evidence on the risks and benefits of cannabis use for ASD. At this time, three large-scale clinical trials are currently at varying stages of progress and publication of results. Only five small studies were identified that have specifically examined cannabis use in ASD. Given the sparse state of evidence directly assessed in this population, studies which examined effects of cannabis on shared pathological symptoms of ASD such as hyperactivity, sleep disorders, self-injury, anxiety, behavioral problems, and communication were also reviewed.

Studies revealed mixed and inconclusive findings of cannabis effects for all conditions, except epilepsy. Adverse outcomes were also reported, which included severe psychosis, increased agitation, somnolence, decreased appetite, and irritability. In addition, a wide range of cannabis compositions and dosage were identified within the studies, which impact generalizability.

There is currently insufficient evidence for cannabis use in ASD, which creates an urgent need for additional large-scale controlled studies to increase understanding of risks and benefits and also to examine the impact of “entourage effects.” This will support discussions of treatment options between health care providers and ASD patients and their families. Evidence may lead to a desired new line of treatment or prevent adverse outcomes from unsubstantiated use amongst families aiming for symptom reduction.

## Background

Cannabis is derived from *Cannabis sativa*, one of the world’s oldest propagated plants. Beginning in the nineteenth century, medical practitioners began to experiment with cannabis to treat tetanus, convulsive diseases, and mental disorders [1, 2] and later, cannabis extracts were available for purchase at physicians' offices and pharmacies, in America and Europe, to treat ailments such as stomach aches, migraines, and insomnia [3].

Today, however, cannabis, which is also commonly referred to as marijuana, remains illegal under federal law in the United States and is categorized as a schedule 1 drug under the Controlled Substances Act. At the state level, cannabis for medical purposes has been decriminalized in over 34 states [4], although physicians remain hesitant in recommending its use given the sparse state of evidence regarding its efficacy to treat specific conditions [5].

Hence, a conflicting spiral exists. Without scientific evidence to establish efficacy, cannabis as a potential course of treatment is often not recommended by practitioners. In turn, until the status of cannabis is changed from a schedule 1 drug, research on the potential uses of marijuana and its components is greatly inhibited [5].

### Medicinal uses of marijuana compounds

The cannabis plant comprises numerous active chemical compounds, which include cannabinoids, terpenoids, and flavonoids. Two cannabinoids include cannabidiol (CBD) and Δ9-tetrahydrocannabinol (THC) [6]. THC is the compound shown to have intoxicating effects and targets the endocannabinoid system in the central nervous system. It affects appetite, cognitive function, memory, and anxiety. CBD on the other hand, is thought to be anti-inflammatory, treat epilepsy and psychiatric disorders, and does so without the intoxicating side effects [7, 8].

Although extensive literature exists on the major cannabinoids CBD and THC, interest has been increasing in other phytotherapeutic compounds of the cannabis plant, specifically, terpenoids. Terpenoids are the fragrant oils, which are naturally present in many plants, and over 200 have been reported. Examples of these include phytol, limonene, nerolidol, myrcene, caryophyllene oxidate, pinene, β-caryophyllene, and linalool. These terpenoids are Generally Recognized as Safe as food additives by regulatory bodies, including the US Food and Drug Administration (FDA), and the Food and Extract Manufacturers Association [6]. Studies in animals and humans have shown the medicinal effects of terpenes’ [9] which demonstrate “anti-inflammatory, antioxidant, analgesic, anticonvulsive, antidepressant, anxiolytic, anticancer, antitumor, neuroprotective, anti-mutagenic, anti-allergic, antibiotic and anti-diabetic attributes” [10]. It is suggested that cannabinoids and terpenes have a combined effect by “working synergistically” with each other. This interaction between the compounds of the cannabis plant is referred to as the “entourage effects,” which have implications on the strains of cannabis which are bred to best treat individual symptoms and diseases [9, 11].

The marijuana plant, however, has not been approved by the FDA for the treatment of any health conditions. Some of its cannabinoids such as CBD, THC, or similar synthetic substances, however, have been approved for specific health issues [12]. At this time, the FDA has approved four drugs with cannabinoids. Epidiolex was approved in 2018 and contains CBD derived from the marijuana plant. It is an oral solution used to treat seizures associated with two rare, severe forms of epilepsy. In addition, Dronabinol and Nabilone, derived from synthetic cannabinoids were approved to treat nausea and vomiting resulting from chemotherapy. Dronabinol is a synthetic form of THC and can be used to aide patients in patients with appetite and weight loss secondary to AIDS. A liquid form of dronabinol, Syndros, was approved in 2016 by the FDA [12]. These medications may offer patients with these ailments an alternative treatment and relief from pain, at a time when a substitute for opioids is crucial [4]. Unfortunately, however, the rising popularity of medical cannabis poses challenges, as evidence to support the use of medical cannabis across many health conditions has been sparse [5].

Based upon the 2018 report from the National Academies of Sciences, Engineering and Medicine (a systematic review of existing studies) strong evidence was reported of the positive effect of cannabis for individuals diagnosed with multiple sclerosis and chronic pain and spasticity, nausea and vomiting due to chemotherapy, and individuals experiencing seizures [13, 14]. However, the report also highlighted sparse or absent evidence supporting medical cannabis for many morbidities, including neurodevelopmental conditions [13, 14], such as autism spectrum disorders (ASD).

The prevalence of ASD has increased and 1 in every 59 children is estimated to be affected [15]. Symptoms such as motor impairment, anxiety, abnormal behavior, sleep problems, and epilepsy substantially impact the quality of life for these individuals [16]. Currently, pharmacological-based treatments attenuate some ASD symptomology, but do not address its underlying etiology [17] although research to examine future pharmacological options is ongoing, such as the interaction of oxytocin and its possible implication in improving social behavior [18].

In the meantime, families of children with ASD are reportedly making cannabis-related decisions based on the plethora of anecdotal evidence on the success of CBD to treat ASD related symptoms and comorbidities [19]. Given the need for additional studies on the effect of cannabis use and the possibility of alleviating symptoms of ASD that substantially interfere with day-to-day work, play, and comfort, it is necessary to review the current state of evidence from human studies to assess the risks and benefits of medical cannabis use amongst this vulnerable population. This will allow positive findings to be noted, highlight adverse health outcomes [20], and consequently identify pathways for future clinical studies.

### The endocannabinoid system

The endocannabinoid system (ECS) is comprised of G-protein coupled cannabinoid 1 (CB1R) and 2 (CB2R) receptors, endogenous bioactive lipid signals (endocannabinoids; eCBs), and both synthetic and metabolizing enzymes [21]. The ECS plays an important role in cannabinergic signaling of human health and disease [22]. The manipulation of eCBs offers therapeutic potential in the treatment and management of a wide range of conditions of the central nervous system, including, but not limited to psychiatric, neurodegenerative, and neuroinflammatory disorders [21, 22].

ASD models in mice have been useful in assessing alterations in the ECS. For instance, Fragile X Mental Retardation (FMR1) knockout mice show core symptoms that are relevant in studies of ASD, including social interaction deficits, repetitive behaviors, and hyperactivity [23–25]. Researchers have identified that alterations in the ECS may be linked to the ASD-like symptoms displayed in the FMR1 model [23, 24, 26]. This points to a potential intervention pathway through modification of ECS signaling, which has shown preliminary success in mouse models in decreasing anxiety and behavioral symptoms, increasing cognitive performance, and attenuating motor deficits.

Numerous bioactive eCBs have been identified, with the most active including Anandamide (AEA) and 2-arachidonoylglycerol (2-AG) [22]. The synthesis of these eCBs occurs via many pathways, including Ca^2+^-dependent N-acyltransferase and N-acylphosphatidylethanolamine-hydrolyzing phospholipase D and diacylglycerol lipase (DAGL) and phospholipase Cβ respectively [21, 27]. AEA and 2-AG are then broken down by fatty acid amide hydrolase (FAAH), and monoacylglycerol lipase (MAGL) [21]. BTBR mice are also used as non-genetic ASD models given that they display anatomical features that are consistent with ASD models [28]. In addition, these mice demonstrate deficits in play, social behavior [29, 30], repetitive behaviors [30, 31], cognitive impairments [32], and excess blood levels of corticosterone in the presence of stressful stimuli [33]. In the BTBR model, increasing AEA levels acute administration of URB597 increased AEA levels, which sunsequently reversed social deficits, though additional studies are needed to investigate altering the ECS in BTBR models [18].

The nature of the ECS is vast and complex, with AEA and 2-AG representing only a couple of the possible eCBs and each having multiple associated pathways of synthesis and enzymatic degradation, with the possibilities multiplying and changing based on regional or tissue-specific locations within the body. Thus, extensive disease-specific research is required into the potential effects of specific eCBs exploitation and modification for therapeutic use [21].

Existing literature highlights potentially promising areas of research, suggesting correlations between the pathogenesis of ASD and the ECS. For example, mutations of neuroligin-3 (a primary protein required for tonic secretion of eCBs) interrupt eCB signaling [34]. Among the effects of such a mutation is the potential for decreased capacity for regulating symptoms of ASDs, such as gastrointestinal function [35–37]. Furthermore, Kerr et al., [38] reported decreased levels of DAGL and MAGL in rats exposed to valproic acid (2-propylpentanoic acid; VPA) Previous studies have found that prenatal exposure to VPA may increase risk of ASD [39] and these findings indicate a possible mechanism by which VPA leads to ASD with respect to altered levels of 2-AG and the corresponding behavioral responses. Additionally, a clinical study completed by Siniscalco et al., [40] demonstrated high expression levels of CB2 in peripheral blood mononuclear cells (PBMCs) of children diagnosed with ASD, indicating the eCBs receptor as a potential target for treatment purposes. Human neuroimaging studies have evaluated the role of the ECS in ASD by measuring responses to social rewards. The studies reported associations between CB1 polymorphisms and ventral striatal cluster activity that suggest a possible link between CB1 polymorphisms and sensitivity to social rewards, a common endophenotype of ASD [41, 42].

### Cannabinoids and its therapeutic mechanism

The mechanism by which cannabinoids could be utilized for treating ASD and its associated disorders, including epilepsy, may possibly be through the synthetic modulation of the ECS, which can help regulate social responses, pleasure, cognition, concentration, body movement, gastrointestinal function, pain, seizures, and the five senses [7, 43]. Unlike THC, CBD is a serotonin (5-hydroxytryptamine) receptor agonist, which is a non-cannabinoid receptor, but may explain the facilitation of the anxiolytic effect [44]. Its antipsychotic effect is attributed to partial agonism at dopamine D2 receptors [45–47], similar to the antipsychotic action of aripiprazole [45]. Additionally, CBD modulates glutamate-GABA systems that may be altered in ASD [48]. Importantly, CBD inhibits the enzyme FAAH that degrades AEA, one of the main endocannabinoids. The modulation of the ECS is primarily targeted to CB1R and CB2R, and synthetic introduction of cannabinoids facilitates a process that mimics natural eCBs signaling to affect physiological factors [49]. THC is more effective in binding to CB1R when compared to binding to CB2R [44]. A high density of CB1R can be found in the basal ganglia, hippocampus, neocortex, hypothalamus, and limbic cortex. These neuron terminals affect motor activity, motor coordination, thinking, appetite, and sedation respectively. CB2R can be found on immune cells and tissues, which affect inflammation and immunosuppression [49], as well as the tonsils and spleen, the central nervous system, and in glial and neuronal cells [50]. These interactions, potentiated by cannabinoid treatment, may offer a prospective treatment option for management of ASD related symptoms in the future. Though CB2R is not expressed in neurons under normal conditions, it is highly expressed under pathological conditions (i.e. psychiatric and neurological diseases) [50], and this warrants further investigation. At this time, although still controversial, immune system dysregulation is beginning to receive attention as having a possible role in ASD [51]. The role of CB2R in regulating the immune system and inflammation offers a potentially promising therapeutic mechanism for managing the symptoms associated with ASD etiology [40, 51]. Previous studies have noted an upregulation of CB2R density and an increase in CB2R protein levels in the PBMC of all of the subjects with ASD, while there were no reported differences in CB1R, nor FAAH levels [40]. No significant intragroup variances were also reported for the control group. These results indicate an endocannabinoid-CB2 signaling dysregulation in ASD, though CB2R has not shown good cannabinoidergic activity [52]. Despite this, there is a hypothesized treatment opportunity for synthetic eCBs manipulation via CBD administration. CBD thus, could offer a therapeutic potential for improving motor skill and sleep, while also supporting anxiolytic, antipsychotic [45], and anticonvulsant symptoms [16].

## Main text

### Methodology

To better understand the current state of evidence regarding the use of cannabinoids among individuals with ASD, we analyzed recently published peer-reviewed literature. Our inclusion guidelines required that an article must be written in English (or a translated text is available), published between the years 2000 and 2019, and focus on cannabinoids in the context of autism spectrum disorders. Academic and publicly-available electronic databases, including the Cochrane Library, MEDLINE, Applied Social Services Index and Abstracts, CINAHL, the Education Resources Information Center (ERIC), EMBASE, and PsycINFO were used as sources of literature that fulfilled the predefined inclusion criteria. Search strategies were customized for each database given its use and depth of controlled vocabulary related to the variables of interest, though “cannabinoids” and “autism spectrum disorder” were the most often used search phrases. As such, systematic reviews, reports, and experimental studies were assessed to understand the nature of the evidence, risks, and benefits of cannabis use for ASD.

### Findings

#### Clinical trials

Clinical evidence to evaluate the benefits, risks, and effects of medical cannabis use for those with ASD, have only just begun. A prospective observational study is currently underway at the Children’s Hospital of Philadelphia, in collaboration with Zelda Therapeutics (NCT03699527), to create a registry of children with ASD who use medical cannabis, follow their natural history of use, and examine the maximum cannabinoid concentrations in pediatric populations with ASD [53].

Since 2016, three clinical trials examining the effects of medical cannabis on individuals with ASD have been undertaken. As part of a larger study, the effect of a single oral dose of CBD versus placebo on the brain of individuals with and without ASD was compared using magnetic resonance spectroscopy [54]. Recently published results from this clinical trial, indicate that “CBD modulates glutamate-GABA systems, but prefrontal-GABA systems respond differently in ASD”. As a result, the authors highlight that the effects of a drug tested in a neurotypical population may not generate similar findings in a population with a neurodevelopmental diagnoses [55].

With a focus on behavioral problems in children and youth with ASD, researchers in Jerusalem are studying the efficacy of a cannibinoid mix, while also examining safety and tolerance. The study is a double-blind randomized placebo-controlled trial and the cannabinoid mix consists of a 20:1 ratio in a 160/8.0 mg per ml of CBD/THC olive oil-based solution [56]. Results from this clinical trial are eagerly anticipated.

A third study is currently ongoing and examining behavioral effects of cannabidivarin (with weight-based dosing of 10 mg/kg/day for 12 weeks) versus placebo on children with ASD. The clinical trial is funded by a $1.3 million grant from the United States Department of Defense [57, 58].

Results from these groundbreaking clinical trials have the potential to help build support for evidence-based recommendations regarding medical cannabis use amongst patients with ASD. Accessing ClinicalTrials.gov is a useful way to follow progress on these studies until the time published results are available. However, additional clinical studies which continue to build on existing evidence remain necessary to fully understand the implications of cannabis use in this population.

#### Preliminary studies

Thus far, only five research studies to the best of our knowledge exist which have examined the direct effects of medical cannabis in individuals with ASD. The most recently published study conducted in Israel, examined the safety and efficacy of medical cannabis use amongst 188 patients with ASD. Most patients were treated using cannabis oil (1.5% THC and 30% CBD), and functional activities of daily living, mood, and quality of life were assessed using structured. Only 93 parents of 155 active participants participated in the six-month follow-up, but a third of participants reported a significant improvement on the three endpoints. Side effects were experienced by approximately 25% of patients, with the most common side effects reported as restlessness followed by sleepiness and psychoactive effects. This study is limited by the follow-up attrituion at the one and six-month follow-up, which was not explained in the publication [59].

In another study also conducted in Israel [8], 53 children with ASD were administered oral cannabinoids under supervision. A 1:20 ratio of CBD and THC was used for a mean duration of 66 days, at a concentration of 30%, with a recommended daily dose of 16 mg/kg for CBD and 0.8 mg/kg of THC (maximal daily dose of 600 mg and 40 mg respectively). The study examined changes in the child’s comorbid symptoms using prospective bi-weekly interviews with parents. Effects of cannabidiol in respect to hyperactivity, sleep problems, self-injury, and anxiety were reported as an improvement, no change, or worsening. Of interest, changes within the cohort for these symptoms was compared to peer-reviewed data for treatment using conventional methods. As such, hyperactivity was considered improved at 80%, self-injury at 82%, sleep problems at 60% and improvement in anxiety symptoms at 64%. Of the children who displayed hyperactivity symptoms, over 68% reported improvement, over 28% had no change, while almost 3% reported worsening of hyperactivity. Improvements in self-injurious behavior were seen in almost 68% of children, 23.5% had no change while almost 9% reported worsening of self-injury. Over 71% reported improvements in sleep, 23.8% had no change, while 4.7% reported worsening effects. Anxiety was improved in over 47% of children, almost 30% had no change, while 23.5% had worse anxiety symptoms. Consequently, the study reported a 74.5% overall improvement in symptoms of ASD comorbidities, although mild adverse effects of somnolence and decreased appetite were reported in 12 and 6 children respectively. The authors reported no statistically significant difference in hyperactivity, sleep or anxiety of cannabidiol oil compared to conventional treatments of these symptoms. Study limitations, however, include lack of an objective assessment tool and a control group [8].

A third study from Israel focused on children with ASD and severe behavioral concerns and assessed the tolerability and efficacy of cannabidiol-rich cannabis. Led by Dr. Aran at the Shaare-Zedek Medical Center in Jerusalem, as a retrospective feasibility study for their clinical trial grant mentioned earlier (NCT02956226) [56], the study systematically assessed 60 children. Participants were prescribed CBD and THC in a 20:1 ratio, as a whole-plant extract dissolved in olive oil ("mean total daily dose was 3.8 ± 2.6 mg/kg/day CBD and 0.29 ± 0.22 mg/kg/day THC for children who received three daily doses (*n* = 44) and 1.8 ± 1.6 mg/kg/day CBD and 0.22 ± 0.14 mg/kg/day THC for children who received two daily doses (*n* = 16)") [60].

The study found 61% of the behavioral problems among participants were “much improved” or “very much improved” according to parent reports. Improvement was also found in anxiety levels in 39% of the children and a 47% improvement in communication. Disruptive behaviors assessed by the Home Situations Questionnaire-Autism Spectrum Disorder [61] and the Autism Parenting Stress Index [62] showed improvement by 29 and 33% respectively. An additional benefit following cannabis treatment was the reduced intake of medications; 24% of participants stopped taking medication, over 30% of children received fewer medications or a lower dose, and 8% received more additional or a higher dose of their current regimen [60].

Despite the fact that promising outcomes were experienced for participants with ASD, adverse events were reported by 57 parents. These side effects most commonly included hypervigilance, which led to worsening sleep concerns (14%), irritability (9%), loss of appetite (9%), and  restlessness (9%). Other frequently cited adverse events included gastrointestinal symptoms, mood changes, fatigue and unexplained laugh. One serious adverse event was reported, with one participant experiencing a transient psychotic event. The study suggests that strains of medical cannabis with a high THC concentration (6:1-CBD to THC ratio) might increase the likelihood of lead to a psychotic state requiring antipsychotictreatment. The uncontrolled retrospective nature of this study has been cited by the authors as a limitation of this study, in addition to the potential for placebo effects reported in controlled treatment studies in children with ASD, as reported by King et al. [60, 63].

A Chilean study published by Kuester et al. [64] examined the effects of cannabis extracts on symptoms of ASD among a small sample of 20 children and one adult with ASD. Participants were monitored after taking sublingual whole plant cannabis extracts for at least 3 months. Almost 72% of the participants used a balanced THC to CBD extract, 19% used a high-CBD option, and almost 10% used high-THC extracts. Details on the administered dosage were not found in the published study or elsewhere; outcomes were assessed using the Clinical Global Impression of Improvement [65] and Autism Parenting Stress Index [62].

Based on these assessments, 66.7% of the participants showed significant improvement in at least one core ASD symptom like repetitive behaviors, language and social communication. Some improvement was reported by most participants including accepting food, sensory difficulties, seizures, and/or sleep disorders. Despite these reported benefits, three patients reported adverse symptoms: increased agitation (*n* = 2) and irritability (*n* = 1). These conditions were resolved with changes to the cannabis strain [64].

The earliest study identified was of a 6-year old male child with ASD conducted in Austria utilizing Dronabinol (THC). The child received THC dissolved in sesame oil with an initial dosage in the morning constituting one drop (0.62 mg) which gradually increased over the 6 months to the maximum tolerated dose of two drops in the morning, one drop midday and three drops in the evening (total dose of 3.62 mg). Significant improvements were noted in hyperactivity, irritability, vocal stereotypy and inappropriate speech symptoms, and sterotypic behavior based on assessments using the Aberrant Behavior Checklist [66] at baseline and after six months of treatment. Hyperactivity dropped by 27 points, lethargy decreased by 25 points, irritability by 12 points, stereotypic behavior by 7 points, and inappropriate speech improved by 6 points [67].

#### Evidence from shared conditions

Although the aforementioned studies illustrate the potential of cannabis to treat core symptoms of ASD, these studies are constrained in their scope of evidence given their small sample sizes, lack of control groups, and other reported limitations. As such, results from the two clinical trials pending publication of results and completion, and additional large scale clinical trials specific to this population will help build evidence for the safety and efficacy of medical cannabinoids for ASD patients. Until this time, evidence for cannabis use in this population can be merely inferred from studies conducted for pathological conditions shared by other patient populations [68]. However, as noted by Pretzsch et al. [55], the inference and transferability of the effects of cannabis treatments from populations without neurodegenerative conditions on the ASD population are speculative.

##### Epilepsy

An estimated 25% of children with treatment-resistant epilepsy (who also display other conditions such as mild to severe intellectual disability, sleep disturbances, mood disorders, and psychosis) are comorbid with ASD [69]. Research on the medicinal use of cannabis for treating individuals with seizures and epilepsy have been extensive and as such, seizure disorders are listed as a qualifying condition in states which permit medical cannabis [70]. Gaston and Friedman [71] discuss the therapeutic mechanism of CBD in treating epilepsy, reporting that rather than targeting CB1R and CB2R, CBD’s anticonvulsant properties target “TRPV1, voltage gated potassium and sodium channels, and GPR55, among others” [71].

An Australian survey conducted by Suraev et al., [72] reported that “15% of adults with epilepsy and 13% of parents/guardians of children with epilepsy were currently using, or had previously used, cannabis products to treat epilepsy. Of those with a history of cannabis product use, 90% of adults and 71% of parents reported success in reducing seizure frequency after commencing cannabis products.”

In an uncontrolled retrospective case study of 272 patients with epilepsy (such as Dravet Syndrome, Rett syndrome, and Lennox-Gastaut syndrome), participants consumed an effective total cannabinoids dose ranging from 0.05 to 9 mg/kg/day with effective serum levels of CBD ranging from 1.8 to 80 ng/ml. Of the participants, 28% of subjects experienced a 76–99% reduction in seizures, 10% experienced a full clinical response, while 14% of participants found no effect of artisanal cannabis preparations in reducing seizures. In addition, increased alertness was reported as a desired side effect, while mild and infrequent side effects included decreased appetite, fatigue and somnolence [70].

Substantial interest and willingness to participate in cannabinoid research has offered a long-awaited potential pharmacotherapy solution to treatment-resistant epilepsy and/or limiting the side effects as compared to other treatments [72]. The literature on cannabinoids and epilepsy, specifically for the treatment of intractable seizures in Dravet and Lennox-Gastaut syndromes and co-occurring autism-like behaviors is, as a result, comprehensive [14] and have led to the recent approval as mentioned earlier, of Epidiolex, an oral cannabidiol [19].

##### Sleep disorders

Problems with sleep is a common comorbidity in children and adolescents with ASD, with prevalance estimated between 40 to 80%. Sleep disorders have a significant impact on these individuals, and affects daily life activities, the ability to interact socially, and have also been associated with increased parental stress [73]. A systematic review by Whiting et al. (2015) assessed the benefits and adverse events of cannabinoids on several diseases and symptoms such as chronic pain, sleep disorders. The review which included 79 trials and over 6400 participants, concluded that there was low-quality of evidence of the effect of cannabinoids on sleep outcomes [74]. In another systematic review conducted by Gates et al. [75], findings suggested that amongst individuals with a medical condition which may impact sleep, the use of cannabinoids could improve sleep through reduced night-time disturbances. However, amongst studies which utilized objective sleep measures, results of sleep outcomes were inconsistent. In one of the studies examined by Gates et al., a double-blind, placebo-controlled-fourway crossover design assessed the effects of cannabis extracts on memory, early-morning performance, sleep, and sleepiness. The four treatments included: “placebo, 15 mg THC, 5 mg THC combined with 5 mg CBD, and 15 mg THC combined with 15 mg CBD, formulated in 50:50 ethanol to propylene glycol and administered using an oromucosal spray during a 30-min period“ at night. Results from the study indicated that 15 mg of THC appeared to have sedative effects while 15 mg of CBD increased alertness [76].

##### Behavioral deficits

An additional core phenotype of ASD is an impaired social functioning ability, including aggression and self-injurious behavior (incidence ranging between 35 and 60%) [68, 77], which can impair academic achievement, education outcomes, rates of employment, and income [2]. Unfortunately, standard treatments do not benefit  approximately 40% of children with ASD and disruptive behavior, leaving caregivers distressed and increasing social isolation [60]. In a review undertaken by the National Academies of Sciences, Engineering, and Medicine, evidence assessed from systematic reviews and clinical studies indicate limited evidence for the link between cannabis use and social functioning [2].

##### Psychosocial and mental health

Anxiety and mood disorders are also commonly reported to affect those with ASD [68], and at least 40% are comorbid with anxiety which aggravates other symptoms [16]. In a double-blind randomized study using healthy controls and patients with social anxiety disorder (SAD) with no previous treatment experience, participants received a placebo or a single administration of CBD (600 mg) one and a half hours before a simulated public speaking test. Participants receiving a CBD dose were noted to have decreased “anxiety, cognitive impairment and discomfort in their speech performance as compared to the placebo group“ [78].

In two studies evaluated by the National Academies of Sciences, Engineering, and Medicine review [2, 79, 80] data was analyzed from waves 1 and 2 of the National Epidemiologic Survey on Alcohol and Related Conditions (*n* = 34,653). Both studies found no association between cannabis use and anxiety disorders, although both studies reported an association between increased cannabis use with an increased odds of SAD (OR, 1.8; 95% CI = 1.1–2.8 and OR, 1.98; 95% CI = 0.99–6.98). The National Academies of Sciences, Engineering, and Medicine based on their systematic and comprehensive review stated that there is limited evidence for the statistical association between the use of cannabis and the development of any anxiety disorder, with the exception of social anxiety disorders. However, there is moderate evidence to support the association between regular cannabis use and social anxiety disorder.

Although less common, psychosis has also been identified as a comorbidity for ASD [81]. As CBD has been shown to have antipsychotic properties in both human and animal studies, an exploratory double-blind parallel-group study was conducted to examine the safety and efficacy of CBD in patients with schizophrenia. Randomized patients were to receive CBD (1000 mg/day) or placebo. If currently prescribed antipsychotic medications, the placebo or CBD was prescribed in addition to the current regiment. CBD may potentially be offered as a new line of treatment for these psychiatric conditions, as “CBD was well tolerated, and rates of adverse events were similar between the CBD and placebo groups” [82]. However, given the adverse outcome of a serious psychotic event discussed earlier in a preliminary study with a patient with ASD [60], the effectiveness of CBD to address psychosis in ASD merits further evaluation.

Effects of cannabinoids on the developing brain of children with and without ASD have also demonstrated the potential for adverse effects such as depressive-like symptoms and an increased risk for psychotic symptoms as an adult [20, 68, 83]. In addition, the impact of cannabis on cognition (specifically, learning, memory, and attention) have also been cited as concerns [2]. Evaluations of studies conducted by the National Academies of Sciences, Engineering, and Medicine illustrate moderate evidence of effects of cannabis on learning, memory, and attention impairment [60, 84], which can impact academic, employment, and social outcomes [2].

Attention-deficit/hyperactivity disorder (ADHD) is also a commonly co-occurring diagnosis in ASD patients with an incidence of 41 to 78% [8]. ADHD also elevates the risk of substance use disorders in children which could complicate the use of CBD for pharmacotherapy in treating ASDs with co-occurring ADHD [85]. An Australian twin study reported “increased liability to ADHD and elevated autistic traits scores were associated with substance use and misuse,” including cannabis use and cannabis use disorders [85].

In a six-week, double-blind randomized placebo-controlled trial researchers assessed the effect of a cannabinoid medication (Sativex Oromucosal Spray) in 30 adults with ADHD on cognition. The treatment comprised of a 100-μl spray, which contained 2.7 mg THC and 2.5 mg CBD. Improvements were demonstrated in hyperactivity/impulsivity, inhibition measures, and a non-significant trend suggesting inattention improvement. One serious adverse event related to muscular seizures and spasms was reported [86].

In addition to these comorbidities discussed above, the effect of cannabis should be examined in light of the possibility of medication  interactions between cannabis and the various prescription drugs individuals with ASD may be utilizing. Research is lacking regarding dosing regimens [14, 19, 20, 87], which increases the risk of adverse outcomes amongst medical cannabis users.

Toxins such as microbes, heavy metals and pesticides associated with the production of cannabis have also raised concerns. While some studies indicate that CBD has low toxicity in humans and no mutagenic effects [68] other studies suggest toxic contamination may be harmful to the reproductive and developmental system and can cause carcinogenicity and infection [20, 88]. This may be of substantial consideration given the concerns of toxins and its potential association with ASD etiology [89].

## Conclusion

Review of these studies demonstrate the mixed state of evidence with respect to the effects of cannabis on core symptoms of ASD, except for certain types of epilepsy. Given the varying types of studies, populations, cannabis compositions and doses reported in the literature for many shared physiological conditions, the risks and benefits of medical cannabis use in ASD are indirect and insufficient. As a result, medical providers treating individuals with ASD should assess the ethical implications of a cannabis recommendation given the uncertainties associated with its utilization at this time. As such, practitioners should consider behavioral supports accessible to the family and only those pharmacological options which are supported by evidence [90]. Although medical cannabis appears to show promise in addressing core ASD symptoms, evidence-based recommendations are necessary to ensure safety and effectiveness.

Results from the randomized and controlled clinical trials [54, 56, 58] will help inform effective compositions (cannabinoids, terpenes and flavonoids) of medicinal cannabis to target symptoms and diseases [11] which also recognize “entourage effects” [9]. In time, these studies may help guide future recommendations of cannabis prescriptions for individuals with ASD.

While research on medicinal uses of cannabinoids continues to expand and some co-occurring disorders of ASD such as epilepsy have been evaluated extensively with cannabinoids; equivalent evidence is not currently available to evaluate the efficacy of cannabinoids in treating other multiple conditions associated with ASDs. Given reports suggesting a dysfunctional endocannabinoid system in ASD, the pharmacologic potential of CBD to impact the symptoms and comorbidities affecting individuals with ASD is significant [43]. Medical cannabis may provide the urgent support needed to address the unique core symptoms of ASD and improving quality of life. Further research, as a result, is necessitated to understand this line of treatment option and to expand the generalizability of results.

## Data Availability

The data supporting the conclusions of this article are available in the databases discussed in the Methodology section and by searching for the specific sources listed in the references.

## Abbreviations

2-AG: 2-arachidonoylglycerol
ADHD: Attention-deficit/hyperactivity disorder
AEA: Anandamide
ASD: Autism spectrum disorder
CB1: Cannabinoid 1
CB1R: Cannabinoid 1 receptor
CB2: Cannabinoid 2
CB2R: Cannabinoid 2 receptor
CBD: Cannabidiol
DAGL: Diacylglycerol lipase
eCB: Endocannabinoids
ECS: Endocannabinoid system
FAAH: Fatty acid amide hydrolase
FDA: U.S. Food and Drug Administration
FMR1: Fragile X Mental Retardation
MAGL: Monoacylglycerol lipase
SAD: Social anxiety disorder
THC: Δ9-tetrahydrocannabinol
VPA: Valproic acid
PBMC: Peripheral blood mononuclear cell
